# Diabetes and Ischemic Stroke: An Old and New Relationship an Overview of the Close Interaction between These Diseases

**DOI:** 10.3390/ijms23042397

**Published:** 2022-02-21

**Authors:** Carlo Domenico Maida, Mario Daidone, Gaetano Pacinella, Rosario Luca Norrito, Antonio Pinto, Antonino Tuttolomondo

**Affiliations:** 1 Molecular and Clinical Medicine PhD Programme, University of Palermo, 90127 Palermo, Italy; carlodomenico.maida@hotmail.com (C.D.M.); bruno.tuttolomondo@unipa.it (A.T.); 2 U.O.C di Medicina Interna con Stroke Care, Dipartimento di Promozione della Salute, Materno Infantile, Medicina Interna e Specialistica di Eccellenza “G. D’Alessandro” (PROMISE), University of Palermo, Piazza delle Cliniche n.2, 90127 Palermo, Italy; pacinella66@gmail.com (G.P.); rosario94.norrito@gmail.com (R.L.N.); pinto@neomedia.it (A.P.)

**Keywords:** diabetes, stroke, cerebrovascular disease, atherosclerosis

## Abstract

Diabetes mellitus is a comprehensive expression to identify a condition of chronic hyperglycemia whose causes derive from different metabolic disorders characterized by altered insulin secretion or faulty insulin effect on its targets or often both mechanisms. Diabetes and atherosclerosis are, from the point of view of cardio- and cerebrovascular risk, two complementary diseases. Beyond shared aspects such as inflammation and oxidative stress, there are multiple molecular mechanisms by which they feed off each other: chronic hyperglycemia and advanced glycosylation end-products (AGE) promote ‘accelerated atherosclerosis’ through the induction of endothelial damage and cellular dysfunction. These diseases impact the vascular system and, therefore, the risk of developing cardio- and cerebrovascular events is now evident, but the observation of this significant correlation has its roots in past decades. Cerebrovascular complications make diabetic patients 2–6 times more susceptible to a stroke event and this risk is magnified in younger individuals and in patients with hypertension and complications in other vascular beds. In addition, when patients with diabetes and hyperglycemia experience an acute ischemic stroke, they are more likely to die or be severely disabled and less likely to benefit from the one FDA-approved therapy, intravenous tissue plasminogen activator. Experimental stroke models have revealed that chronic hyperglycemia leads to deficits in cerebrovascular structure and function that may explain some of the clinical observations. Increased edema, neovascularization, and protease expression as well as altered vascular reactivity and tone may be involved and point to potential therapeutic targets. Further study is needed to fully understand this complex disease state and the breadth of its manifestation in the cerebrovasculature.

## 1. Introduction

Stroke and diabetes mellitus are two separate conditions which share multiple common threads and contribute to a growing cardiovascular disease burden and mortality around the world. Both are increasing in prevalence, both are diseases which affect blood vessels, and both are associated with other vascular risk factors, such as hypertension and dyslipidemia. Diabetes mellitus is an established risk factor for stroke and maybe associated with poorer outcomes after stroke. Indeed, abnormal glucose regulation, of which diabetes is one manifestation, is seen in up to two-thirds of people suffering from an acute stroke and make diabetic patients more likely to die or be severely disabled and less likely to benefit from the one FDA-approved therapy, intravenous tissue plasminogen activator. On the other hand, cerebrovascular complications make diabetic patients 2–6 times more susceptible to a stroke event and this risk is magnified in younger individuals and in patients with hypertension and complications in other vascular beds. On this premise, the aim of this narrative review is to analyze the current evidence from literature supporting the strict association between cerebrovascular diseases and ischemic stroke. The literature search has been conducted through PubMed Central/Medline and Embase through January 2022.

## 2. Diabetes Mellitus and Atherosclerosis: Vascular Complications

### 2.1. Definition of Diabetes Mellitus

‘Diabetes mellitus’ is a comprehensive expression to identify a condition of chronic hyperglycemia whose causes derive from different metabolic disorders characterized by altered insulin secretion or faulty insulin effect on its targets or often both mechanisms [1].

Other types of diabetes can be classified according to the pathophysiological mechanism:

- Type 1 diabetes (T1D), characterized by pancreatic β-cell destruction with absolute insulin deficiency, usually caused by immunological disorders (this form includes LADA, latent autoimmune diabetes of the adults, with late-onset);
- Type 2 diabetes (T2D), with a broad spectrum of variations ranging from the prevailing insulin resistance with relative insulin deficit to predominant insulin secretion defect with insulin resistance;
- Gestational diabetes, connoted by the occurrence of diabetes during pregnancy and its resolution at the end of the gestational period, though some complications developed in this phase may be irreversible;
- Other specific diabetes types (including disorders of the exocrine pancreas like pancreatitis or cystic fibrosis, endocrinopathies like Cushing syndrome or pheochromocytoma, drug-induced by glucocorticoids or neuroleptics, genetic forms of β-cell dysfunction-MODY, genetic syndromes with altered glycemic metabolism, and infectious diseases) [2].

The pathophysiological mechanisms of diabetes mellitus, as mentioned above, are the result of the alteration of the mechanisms that regulate blood glucose levels: on the one hand, insulin resistance causes an increase in glucose production in the liver, reduced storage capacity in the form of glycogen and reduced utilization by muscle tissue; on the other hand, β-cell dysfunction causes reduced insulin production and consequent dysregulation of glucose regulation mechanisms. The presence of these alterations, and sometimes a combination of them, forms the basis for the onset of the disease. When these dysregulations are present, β-cell dysfunction is more severe than insulin resistance and causes a more rapid evolution towards the onset of T2D [3,4].

The damage to β-cells in T2D has always been attributed to cell death, but evidence suggests that the dysfunction is due to a complex network of molecular alterations to which pancreatic cells are subjected. In a condition of nutrient excess (typical, for example, of obesity), hyperglycemia and hyperlipidemia predispose to oxidative stress and a consequent chronic inflammatory state that causes a loss of integrity of the pancreatic islets [5].

An excess of free fatty acids and glucose causes damage to the endoplasmic reticulum through the activation of apoptotic unfolded protein response (UPR) pathways [6]: several mechanisms alter cellular homeostasis, such as the inhibition of sarco/endoplasmic reticulum Ca2+ ATPase (SERCA) responsible for the mobilization of intracellular calcium and the activation of inositol 1–4-5 triphosphate (IP3) receptors; moreover, hyperglycemia causes an increase in the synthesis of proinsulin and islet amyloid polypeptides (IAAP) in the β-cells, resulting in an accumulation of misfolded insulin and proteins damaged by oxidative stress already mentioned. All these processes compromise the physiological mobilization of calcium from the endoplasmic reticulum, activate pro-apoptotic signals, determine degradation of the insulin mRNA and promote the release of IL-1β, which triggers inflammation of the pancreatic islets [7]. The result of this is the loss of the physiological communication between the cells and the alteration of the feedback mediated by insulin and glucagon, resulting in hyperglycemia that feeds and perpetuates the process.

Of course, as with any pathology, in addition to promoting environmental factors, we need to consider the genetic basis within which diabetes develops: genome-wide association studies, in recent years, have documented the polygenic nature of T2D and have made it possible to identify several loci with genes involved in insulin secretion and the onset of insulin resistance [8,9]; the data available today are still limited and need to be expanded, but a better understanding of the genes involved in the onset of diabetes could undoubtedly be helpful in characterizing the pathology better and to choose the most appropriate therapy for each patient, giving specific weight to risk factors and predisposition.

Indeed, a knowledge of the molecular mechanisms that lead to the onset of pathology has practical repercussions on the choice of therapy, considering the large availability of drugs with very different mechanisms capable of acting, selectively, on the most implicated metabolic pathway: starting with insulin-sensitizing drugs, passing through the analogues of the incretin hormones, arriving at the inhibitors of sodium/glucose co-transport; from this assumption it is possible to modify the cardiovascular risk of diabetic patients, obtaining a strategy ‘sewn on the patient’ and this, together with changes in lifestyle and eating habits, can allow us to reduce the weight of this public health issue substantially.

T2D has become more frequent in recent years in every country globally. This critical and progressive growth can depend on various causes: the ageing of the world’s population, the massive economic development of industrialized countries, the consequent change in eating habits, sedentary lifestyle.

However, because of the lack of symptoms and signs in the early stages of the disease, the diagnosis of T2D is made when complications have already occurred: usually, this condition becomes evident when the patient has angina pectoris, myocardial infarction, ischemic stroke, PAD (peripheral artery disease), or heart failure [10].

The prevalence of T2D has increased considerably in recent decades. For example, there were 108 million patients in 1980 (4.7% of the population), and this number has grown enormously over 40 years (425 million, 8.5% of the people in 2017).

It is estimated that in 2065 there will be 629 million patients with diabetes; this confirms that the epidemiological weight of the disease is far from secondary [11].

The current diagnostic criteria of T2D were formulated in 1997 when the first Expert Committee on the Diagnosis and Classification of Diabetes Mellitus examined data from three cross-sectional studies that had evaluated the correlation between retinopathy and blood glucose concentration as FPG (fasting plasma glucose), 2-h PG (2-h plasma glucose), and HbA1C (glycated hemoglobin): through this analysis, was detected the glycemic threshold below which retinopathy was little prevalent and, on the base of this information, was fixed a new diagnostic cut off of ≥126 mg/dL (7 mmol/L) for FPG and was kept the precedent long-standing 2-h PG value of ≥200 mg/dL (11.1 mmol/L) for diagnosis of T2D [12].

HbA1C is an essential marker of diabetes mellitus because it reflects average blood glucose concentration over 2–3 months: it is widely used because it correlates with both micro and macrovascular complications of diabetes and demonstrates the adequacy of glycemic management, allowing therapeutic modifications to improve the patient’s outcome.

However, the clinicians did not use HbA1C to diagnose T2D in the past because of the lack of standardization of assays. Nowadays, the International Expert Committee has recommended, based on epidemiological evidence, the use of HbA1C to diagnose diabetes with a threshold of ≥6.5%, and the ADA (American Diabetes Association) confirmed this decision [13].

There is a ‘grey area’ between the physiological glucose metabolism and diabetes mellitus, a condition known as ‘prediabetes’. Prediabetes is an alteration of glucose homeostasis characterized by impaired fasting glucose (IFG, fasting plasma glucose 100–125 mg/dL) below the diagnostic threshold for diabetes, impaired glucose tolerance (IGT, i.e., 2-h plasma glucose 140–199 mg/dL after a load of carbohydrate) or both these conditions. Therefore, it is necessary to obtain fasting glucose levels after an overnight fast of 8 h; it is also crucial that the patient follows precise rules to not affect glycemic metabolism the day before the test. According to the recent international guidelines, glycated hemoglobin (HbA1C) should be used to determine the condition of prediabetes with the cutoff of 5.7% to 6.4% [14].

The actual burden of T2D is due to daily care and various disease complications. Although the common factor is the high blood glucose serum level, different pathophysiological mechanisms are at the base of macrovascular, microvascular, and neurologic disorders determined by diabetes. Macrovascular complications refer to atherosclerosis of coronary and legs arteries: elevated glucose levels—with other risk factors like hypercholesterolemia, smoking, and sedentary lifestyle—can generate alterations in platelet function and fibrin, increasing thrombosis and degeneration the atherosclerotic plaque. Microvascular disorders are related to endothelial damage of the capillaries and involve mainly the anatomical district of eyes and kidneys: diabetic retinopathy is the most common cause of visual impairment and blindness in adults, renal involvement and its progression to an end-stage renal disease requiring dialysis are very frequent [15].

The particular element of vascular complications in diabetes mellitus is that the free passage of glucose into the cells characterizes nerve and vascular tissues. Insulin is necessary to regulate the internalization of glucose in other tissues. Still, in vascular endothelium and nerve cells, this mechanism is absent, and in these cells, the glucose level is the same as the plasma. The excess of glucose inside these tissues activates disposal processes and alternative pathways of metabolism (glycosylation of substrates, polyol pathway, etc.) that damage the cell membranes and their functioning, leading to the complications described above [16].

Beyond the alterations of the glucosidic metabolism, diabetes mellitus is considered a systemic metabolic disease because of the dysregulation of lipidic metabolism: insulin action on adipocytes and free fatty-acids levels are altered in these patients so the impairment of the lipid homeostasis and the vascular damage produced by the mechanisms already described increase the cardiovascular risk and death for cardiovascular causes [17].

These alterations, also considering the endocrinological role of adipose tissue, cause a subclinical chronic inflammation: in the natural history of diabetes is possible to detect an increased level of inflammatory biomarkers, produced mainly by adipocytes. The activation of inflammation pathways in diabetic patients suggests an essential role of the inflammatory environment in developing vascular complications and clarifies the link between DM and cardiovascular diseases [18].

### 2.2. Definition of Atherosclerosis

Atherosclerosis (ATS) is the most frequent cause of arterial vasculopathy and is undoubtedly an insidious condition: it is unlikely to be the trigger in coronary artery disease, ischemic stroke, and peripheral artery disease (PAD) on its own; instead, it acts together with other chronic degenerative diseases such as arterial hypertension and diabetes mellitus to constitute the physiopathological basis of cardio- and cerebrovascular accidents [19].

ATS results from a combination of some events, including hyperlipidemia and endothelial injury, and its typical feature is the intimal plaque [20].

The deposit of lipids in the inner layer of the vascular wall triggers the development of atherosclerotic plaque; later, fibroblasts and smooth muscle cells produce a fibrous cap. The growth of plaque leads to a reduction in blood flow to the anatomical districts vascularized by the affected arteries and, among other things, the possible exposure of the lipid core favor thromboembolic phenomena that can affect virtually any organ [21].

As mentioned above, endothelial cells, leukocytes, and smooth muscle cells are the protagonists of the atherosclerotic process.

Atherosclerotic lesions begin to occur in areas where the endothelium is dysfunctional and damaged. In these areas of increased susceptibility, depending on the plasma concentration of lipoproteins, there is leakage into the subendothelial space where the potentially atherogenic lipoproteins are oxidized, becoming cytotoxic pro-inflammatory, and chemotactic. The mechanisms by which low-density lipoproteins (LDL) are modified in an atherogenic way are only partly known and certainly include oxidative mechanisms linked to the production of reactive oxygen species and the production of nitric oxide [22].

Nitric oxide (NO) has a different effect depending on the source of its production: NO produced by endothelial nitric oxide synthase (eNOS) has a vasodilating and athero-protective effect. In contrast, the one produced by macrophage inducible nitric oxide synthase (iNOS) has a bactericidal function due to its oxidizing role and its atherogenic impact.

Inflammatory and atherogenic stimuli cause the exposure of adhesion molecules by the dysfunctional endothelium, mainly vascular cell adhesion molecule-1 (VCAM-1), with important recruitment of monocytes and T lymphocytes that play a crucial role in the progression of the atherosclerotic lesion [23].

Several studies have observed that in atherosclerotic plaque, the recruitment of leucocytes is crucial and depends fundamentally on the presence of oxidized LDL and monocyte chemotactic protein-1 (MCP-1). The recruited monocytes differentiate into macrophages and internalize the oxidized lipoproteins through scavenger receptors, the most important of which are SR-A and CD36. Lipid accumulation in macrophages continues until the death of these cells because scavenger receptors, unlike native LDL receptors, do not downregulate in response to the collection of cholesterol esters: cell death by necrosis and apoptosis of histiocytes results in the genesis of a lipid core that causes plaque expansion [22].

Macrophages, in addition to playing a key role in the genesis of plaque, also have a destabilizing function in that they produce proteolytic enzymes capable of degrading the extracellular matrix (metalloproteinases) with exposure of the core and activation of prothrombotic processes: ‘the sentinels of the immune system’, therefore, despite having an innate defence function for the human organism, become the main enemies of the arteries and contribute substantially to plaque degeneration [23].

In the progression of the disease, the immuno-inflammatory response favors the activation of a fibroproliferative mechanism mediated by smooth muscle cells; these cells are responsible for repairing tissue damage after the genesis of the arterial lesion. As the atherogenic stimulus persists over time, the repair process continues, and the plaque increases in size to the point of determining a reduction in the diameter of the vessel, which can compromise downstream flow [24].

Due to their ability to produce the collagen-rich extracellular matrix, smooth muscle cells are considered, to a certain extent, protective against complications related to plaque rupture such as thrombosis. Thus, their recruitment, ability to reproduce and activation are deemed positive for plaque stability, while senescence and apoptosis are risk factors: it has been observed that there is a depletion of these cells at plaque rupture sites [25,26,27].

On the other hand, T-cells release tumor necrosis factor-alpha (TNF-α), which inhibits collagen production by smooth muscle cells, and this, together with the production of metalloproteinases by macrophages, cause degradation of the extracellular matrix. These mechanisms lead to plaque’s instability and possible rupture, causing thrombosis and acute ischemia [28].

The term ‘plaque rupture’ is used to identify damage to the fibrous cap leading to exposure of the lipid core [29]. This process causes surface hemorrhage of the plaque and thrombotic phenomena for platelets activation, which is responsible for the sudden and silent progression of the lesions [30].

A recent meta-analysis showed that ruptured atherosclerotic plaques cause 76% of fatal heart attacks; the remaining 24% are caused by plaque erosion and other mechanisms that are not yet fully defined [31].

Atherosclerosis, as already mentioned concerning the role of oxidized LDL, is an inflammatory disease: monocytes that differentiate into macrophages as they migrate towards the forming plaque, release significant quantities of inflammatory cytokines at the site of the lesion—such as interleukin-6 (IL-6) and TNF-α—which are responsible for stimulating the liver to produce C-reactive protein (CRP). In addition, under conditions of prevalent pro-inflammatory stimuli, adipose tissue may also produce CRP [32].

The production of CRP, in the subject with significant atherosclerotic burden, represents a vicious circle: it is the inflammatory basis for which atherosclerotic damage sets in, but it is also the consequence of the amplification of vascular damage, which is made even worse by the spread of the inflammatory microenvironment.

For this reason, it seems that serum CRP levels are helpful for diagnosis, response to treatment and for assessing exposure to atherosclerotic stimuli. Moreover, several studies have shown that CRP binds to LDLs and is present in plaques, becoming detectable even in the early stages of atherogenesis. For this reason, it is regarded as a predictor of future cardiovascular events to the extent that serum CRP levels are independent risk factors for all-cause mortality [33].

### 2.3. Vascular Complications of Diabetes Mellitus

T2D and atherosclerosis are, from the point of view of cardio- and cerebrovascular risk, two complementary diseases. Beyond shared aspects such as inflammation and oxidative stress, there are multiple molecular mechanisms by which they feed off each other: chronic hyperglycemia and advanced glycosylation end-products (AGE) promote ‘accelerated atherosclerosis’ through the induction of endothelial damage and cellular dysfunction.

These diseases impact the vascular system and, therefore, the risk of developing cardio- and cerebrovascular events is now evident, but the observation of this significant correlation has its roots in past decades.

There have been several findings of experiences in which this correlation seems to be evident in scientific literature. For example, in 1969, Heyden et al. reported retrospective case-control studies and prospective studies from Los Angeles and Framingham, in which it was apparent that hypertension and DM were risk factors for cerebrovascular diseases and that the early treatment of DM could be essential to prevent or delay the onset of stroke [34].

Over the years, it has become clear that the role of T2D is decisive in triggering cardio and cerebrovascular events: in 1977, Królewski et al. made a retrospective analysis through which observed, in diabetic patients, a mortality rate 1.3 times higher than in non-diabetic subjects and noticed that the causes of death were mainly coronary heart disease and cerebrovascular disease. Furthermore, the risk of death for cardiovascular diseases was greater in patients with early onset of diabetes, as evidence that the duration of the disease and its pathophysiological mechanisms are connected with vascular disorders. Moreover, these observations were possible because of the discovery of insulin and its use for the treatment of diabetes: this drug prolonged the life expectancy of diabetic patients by reducing acute complications, such as acidotic coma, and made it possible to observe vascular complications over time [35].

Although diabetes was often considered a risk factor for myocardial infarction or ischemic stroke in the context of an atherogenic profile, several scientific works have shown the role of T2D as an independent risk factor for cardio and cerebrovascular diseases. For example, the Honolulu Heart Program, a prospective study of cardiovascular disease, assessed the risk of stroke considering two cohorts of patients (diabetic patients vs. non-diabetic subjects without coronary heart disease and history of stroke at the beginning of the study). The relative risk of having an ischemic thromboembolic stroke for diabetic patients compared with non-diabetic subjects was 2.0 (95% confidence limit, 1.4 to 3.0), and it was observed that tight control of other atherogenic conditions (i.e., hypertension, hypercholesterolemia, sedentary lifestyle) did not decrease the effect of diabetes in causing the stroke. Therefore, diabetes mellitus appears to be an additional independent risk factor for ischemic stroke (no correlation between diabetes and hemorrhagic stroke was observed) [36].

The significant impact of vascular diseases in diabetic patients has become increasingly important: it is now clear that diabetic people have an increased risk of coronary heart disease, peripheral arterial occlusive disease, and death for cardiovascular causes compared with non-diabetic subjects. The Multiple Risk Factor Intervention Trial, in 1993, showed this aspect more sharply: the incidence of cardiovascular diseases and CHD (coronary heart disease) mortality increases in both diabetic and non-diabetic cohorts of patients as cholesterol levels rise, but the mortality rate among people with diabetes is four or five times higher than non-diabetics at the same cholesterol levels [37].

The correlation between the duration of diabetes and the risk of cerebrovascular disease has become progressively evident. For example, it has been found that among patients with stroke younger than 55 years, those with diabetes mellitus had a risk more than 10-fold [38].

To confirm the above, the experience of the Baltimore-Washington Cooperative Young Stroke Study is crucial: were enrolled 296 cases of ischemic stroke among young adults from 18 to 44 years and, through this analysis, the study showed that diabetes increased the odds ratio for stroke, from 3.3 for black women to 23.1 for white men evidence that, in addition to gender differences, diabetes plays a predominant role in the risk profile for stroke [39].

Patients with diabetes of all age groups have a probability at least twice of stroke compared with non-diabetic subjects. In addition to the duration of diabetes, there is another factor to evaluate the prognosis of diabetic patients with stroke: they have an elevated risk of a future ischemic event. The close connection between DM and ischemic stroke, therefore, is not only due to the comorbidities of diabetic patients who have a cerebrovascular disease (i.e., hypertension, hypercholesterolemia) but also to diabetes-specific properties involving small penetrating arteries: people with diabetes have a significant probability of suffering a lacunar stroke or small vessel disease [40].

## 3. Molecular Pathology of Vascular Damage in Diabetes Mellitus and Atherosclerosis

The correlation between diabetes mellitus and ischemic stroke has been found by several authors and is a common experience in clinical practice. In agreement with previous studies, Matz et al. discovered a high prevalence of glucose metabolism disorders in patients with ischemic stroke, including new-onset diabetes mellitus. While diabetes is a known risk factor for the occurrence of ischemic stroke, it remains to be understood whether asymptomatic hyperglycemia has a potential role in causing a cerebrovascular event [41,42].

The frequency of T2D is three times higher in patients with ischemic stroke than in controls [43].

Indeed, the risk of stroke increases from 150% to 400% in diabetics, and the extent of glycemic dyscontrol correlates directly with the risk of acute cerebrovascular accidents [41,44].

The altered metabolic framework that characterizes diabetes mellitus influences vascular changes. The most characteristic abnormalities certainly include chronic hyperglycemia, insulin resistance, and dyslipidemia: these factors can promote the atherosclerotic structure and induce cellular dysfunction at various levels (like endothelial dysfunction, smooth muscle cell alterations, platelet abnormalities, and coagulation alterations).

Concerning endothelial dysfunction, it should be remembered that endothelial cells represent the inner lining of the vascular lumen and perform, in addition to the mechanical function of coating, an endocrine role through the production of biologically active substances that regulate various functions: nitric oxide, prostaglandins, endothelin-1, angiotensin II, and other reactive oxygen species. Nitric oxide dilates the vessels, inhibits platelet activation, reduces the proliferation of smooth muscle cells, and reduces the process of diapedesis of leukocytes. The combination of these phenomena aims to limit the atherosclerotic pattern, ensuring the vascular system’s integrity [45,46,47].

Several studies have shown that T2D alters endothelial function through a cascade of molecular events that precede atherosclerotic plaque formation but promote its appearance. Constant hyperglycemia inhibits the enzyme eNOS (endothelial nitric oxide synthase) and thus cause the reduction of nitric oxide and the stimulation of the production of reactive oxygen species, including superoxide anion (O^2−^) [48]; superoxide anion neutralizes nitric oxide by producing the toxic ion peroxynitrite, which uncouples the eNOS enzyme by oxidizing its cofactor, tetrahydrobiopterin [49].

Another mechanism that limits the production of nitric oxide is related to insulin resistance: the reduced action of insulin on adipocytes leads to an increased release of free fatty acids from adipose tissue [50] with consequent activation of the pathway of protein kinase C, then inhibition of phosphatidylinositol-3 (PI-3) kinase and increase of reactive oxygen species at the expense of nitric oxide [51].

While, from what has been said above, T2D reduces the production of mediators of vasodilation, it is also true that in diabetic patients, the production of vasoconstrictive substances increases, including, crucially, endothelin-1.

Endothelin-1 has a dual effect: it promotes vasoconstriction through its action on smooth muscle and activates renal salt and water retention, resulting in activation of the renin-angiotensin-aldosterone system and thus smooth muscle hypertrophy [52].

The concentration of endothelin-1, among other things, increases in response to the insulin-mediated effects of increased gene expression and receptor synthesis and as a consequence of the increased presence of glycosylation products, so its vaso-active effects in diabetes are multiple and complex [53].

Moreover, endothelial cells regulate the cell transit through the vessel wall producing chemotactic adhesion molecules: monocytes, once reaching the subendothelial space, phagocytize oxidized LDL and become foamy cells, the initial substrate of atherosclerotic lesions [23].

**Figure 1:**
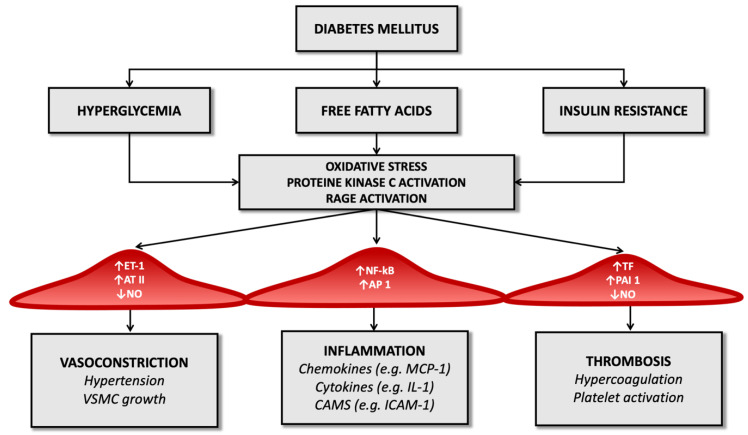
Diabetes’ mechanisms of vascular damage.

Advanced glycosylation end-products (AGEs) also amplify endothelial cell damage: high serum glucose levels produce a process of glycation and glycosylation of proteins at the extracellular level, leading to their accumulation. These molecular products cause the increased expression of adhesion molecules on endothelial cells, promote the migration of monocytes/macrophages towards the forming atherosclerotic plaque and promote the release of inflammatory cytokines by histiocytes. They also contribute to changes in the extracellular matrix and are a risk factor for plaque rupture [54,55] (Figure 1).

In the diabetic patient, therefore, all these molecular pathways (hyperglycemia, increased oxidative stress, and activation of receptors for advanced products of glycosylation) promote the expression of the gene for the nuclear factor kappa-light-chain-enhancer of activated B cells (Nf-kB): this leads to increased production of inflammatory mediators (such as interleukin-1) and adhesion molecules for leukocytes that favor atherogenesis [17,56,57].

Thus, endothelial dysfunction represents a significant factor in the pathogenesis of ischemic stroke and is a shared effect of diabetes mellitus and atherosclerosis, the ‘accelerator pedal’ on which both these diseases press.

The reduced bioavailability of nitric oxide and the prevalence of molecules mediating vasoconstriction in people with diabetes results in altered smooth muscle cell function: infusion of angiotensin II or endothelin-1, in fact, in these patients, results in a lower vasoconstriction effect than in healthy controls [58,59,60].

Among other things, diabetic patients have autonomic dysfunction that modifies peripheral vascular resistance through mechanisms that are not yet fully known and this, together with the above, constitutes an essential element of vascular damage [61].

The altered metabolic pattern generated by diabetes also results in structural and functional alterations in smooth muscle cells. In non-diabetic subjects, during the atherogenic process, muscle cells migrate from the intermediate layer of the vascular wall to the forming atherosclerotic plaque, replicating and contributing to the extracellular matrix’s constitution. In people with T2D, an increased migration capacity of smooth muscle cells has been demonstrated in vitro. Still, these cells are reduced in complicated atherosclerotic plaques [62] since hyperglycemia determines modifications on the oxidation process of low-density lipoprotein (LDL) that favor their apoptosis [63].

The progression of atherosclerosis and the risk of plaque rupture in diabetic patients is also linked to platelet dysfunction generated by the disease: the concentration of glucose inside platelets, like endothelial cells, does not depend on the action of insulin but the extracellular concentration of glucose. The increased intracellular availability of glucose causes activation of protein kinase C, reduced nitric oxide production, and increased reactive oxygen species, as mentioned above about endothelial cells [64].

The result is impaired platelet function, altered homeostasis of intracellular calcium, and dysregulation of the thromboxane synthesis process. In addition, in diabetic patients, increased surface expression of glycoprotein Ib (which mediates interaction with von Willebrand factor) and glycoprotein IIb/IIIa, which regulates interaction with fibrin and leads to a significant increase in potential thrombotic risk [65].

Platelet alteration is only one aspect of the alterations inherent in the coagulation process. For example, several studies have observed that in diabetic patients, there is reduced fibrinolytic activity due to the high concentration of the tissue plasminogen activator inhibitor type 1 both in atheromatous plaques and in arteries not affected by the atheromatous process [66].

Diabetes also determines an imbalance between factors that regulate the hemostatic balance, favoring the increased availability of tissue factor and factor VII of the coagulation cascade (which have a potent procoagulant activity) and determining a reduction in serum levels of anticoagulant factors such as protein C and antithrombin III (Figure 1). It seems that these alterations depend directly on the condition of hyperglycemia that characterizes T2D and partly on the cleavage products of proinsulin in the process of transformation to insulin [67].

Chronic inflammation is another determinant of thrombotic risk and is a common feature of atherosclerosis and diabetes mellitus. Increased inflammasome activity and high levels of nucleotide-binding oligomerization domain-like receptor 3 (NLRP3) have been documented in diabetic patients, together with increased serum levels of pro-inflammatory cytokines such as interleukin-1 beta and interleukin-18. One of the common aspects of diabetes and atherosclerosis in the pattern of inflammation is neutrophil extracellular trap activation, or NETosis, a particular type of cell death in macrophages through which chromatin is released into the extracellular space to trap and kill bacteria. This mechanism is typical of chronic inflammatory conditions and infections, and it has been observed that it can be promoted by hyperglycemia [68].

Additionally, in animal models, NETosis has been shown to promote atherosclerosis so that, through anti-diabetic therapy, it is possible to limit the atherosclerotic burden by defining the process of atherosclerotic plaque genesis.

In addition to the already known mechanisms by which T2D promotes inflammation, recently, the high-mobility group box 1 protein (HMGB1), non-histone proteins that act as an alarm for the immune system to initiate tissue repair and host defence processes, have been increasingly studied. It appears that during the acute phase of stroke, these proteins migrate towards the extracellular space and are bound by Toll-like receptors (TLR-2, TLR-4) and receptors for advanced glycosylation end-products (RAGE) that activate the transcription factor Nf-kB and the synthesis of inflammatory cytokines that worsen outcome and prognosis [69].

According to [60], in diabetic patients, the excess presence of advanced glycosylation end-products (AGEs) and their receptors (RAGEs) is an unfavorable element, so the possibility of limiting the expression of HMGB1, especially in diabetics, is being investigated to limit the inflammatory pattern of ischemic stroke and to improve the prognosis of these patients.

Altogether, diabetes mellitus is a true generator of thrombotic risk and, therefore, a promoter of ischemic stroke: on the one hand, it activates molecular damage mechanisms leading to endothelial dysfunction and the progression of the atherosclerotic process, and on the other, it increases the thrombogenic risk through the induction of platelet dysfunction and the dysregulation of the coagulation cascade.

## 4. Epidemiology of Ischemic Stroke in T2DM Patients

Type 2 diabetes mellitus (T2DM) is one of the most common chronic pathologies, and in 2015 nearly 400 million subjects in the world were considered diabetic; the prevalence of this disease is supposed to increase up to 640 million people in the next 20 years [70].

Stroke affects more than 500,000 persons every year in the United States and represents one of the most significant reasons for decease in the Western part of the world [71]; in addition, ischemic stroke is the second most frequent complication of T2DM after coronary artery disease (CAD) [72], and the second source of death after cancer in diabetic subjects [73].

The Framingham Study showed that the incidence of ischemic stroke in diabetic patients was 2.5–3.5 times increased compared to the control group [74]. Similar results have been observed in multiple research [75,76].

While cardioembolic stroke is more common in non-diabetic subjects, diabetes is correlated to cerebral ischemia caused by atherosclerosis [77]; the endothelial dysfunction can easily explain this due to the persistent hyperglycemia and by the concomitant presence of other risk factors for atherosclerosis, such as hypertension and hyperlipidemia.

In an observational study, Mulnier et al. reported a rate of stroke of 11.9 per 1000 persons per year in subjects affected by DM, in the healthy group, this rate was 5.5 per 1000 person-year; the highest hazard ratio correlated with ischemic stroke was noticed in the 35–54 year group, in addition, the risk was significantly higher in women than in men [78]. As observed by Selvin et al., there is a correlation between the levels of glycated hemoglobin (HbA1c) and the chance of cerebral ischemia [79]. Furthermore, proteinuria (>300 mg/dL) has been associated with an increased risk of ischemic stroke, even if it does not seem to influence the prognosis [80].

T2DM is rarely an isolated condition; in the majority of the cases, it is associated with other well-known risk factors for ischemic stroke, such as dyslipidemia and hypertension. Consequently, it is necessary to operate on these pathologies to reduce the chance of cerebral ischemia in diabetic subjects.

Kearney et al. performed a meta-analysis of 14 randomized trials including more than 18,000 subjects affected by diabetes mellitus; statin therapy led to a significant reduction of the risk of ischemic stroke; this reduction was more marked in diabetic patients than in the control group [81].

As far as it concerns hypertension, the UK Prospective Diabetes Study (UKPDS) showed that the reduction of 10 mmHg in systolic blood pressure resulted in a more than 40% decrease in stroke incidence [82]. Furthermore, treatment with indapamide plus perindopril was associated with a 38% diminution of the stroke risk in patients affected by diabetes mellitus, as reported by the Perindopril Protection Against Recurrent Stroke Study (PROGRESS) [83].

Moreover, Lichtman et al. reported diabetes as an independent risk factor for cerebral ischemia in the first six months after acute coronary syndrome [84]; the same finding was observed after coronary artery bypass grafting [85].

Diabetes mellitus is a common comorbidity in subjects affected by an ischemic stroke. Up to 20% of stroke patients have diabetes [86,87,88]; furthermore, Gray et al. reported a new diagnosis of diabetes by 12 weeks after the stroke in nearly 20% of the subjects [89].

Multiple studies reported the significant association between DM and lacunar stroke [90]. This finding is quite plausible because hyperglycemia, together with hypertension, plays a crucial role in the process of lipohyalinosis, which is the leading cause of small vessel disease. Nevertheless, some studies did not find this association [91,92].

## 5. Most Common Stroke Subtypes in Diabetes

It has been well established that diabetes represents one of the most critical risk factors for ischemic stroke (Table 1). These data have been widely described in several studies since 1979 in the Framingham study [74], based on 20 years of cohort surveillance, in which the authors described a 2.5-fold incidence of ischemic stroke in diabetic men and a 3.6-fold one in diabetic women.

Similar data have been shown in other extensive studies, Manolio et al. in the 1996 and Giles et al. in 1995 [93,94], where the authors analyzed a large population of respectively 5201 and 9565 people, and reported, odds ratio (OR) of 2.12 and 2.47 in those who have diabetes after an adjustment for other risk factors.

This increased risk has been linked to the pathophysiological changes seen in the cerebral vessels of patients with diabetes, and the mechanisms by which hyperglycemia and diabetes determine the onset of ischemic stroke are different and have been previously mentioned.

However, it must be stressed that stroke is a heterogeneous disease, with different subtypes due to different etiopathogenetic mechanisms, different sites and sizes of infarction, and different outcomes regarding disability and risk of death. For this purpose, several authors have tried to identify whether diabetic patients with stroke shared clinical and phenotypic characteristics or represented a peculiar category compared to those who had a stroke due to other canonical cardiovascular risk factors such as hypertension and dyslipidemia.

Megherbi et al., in a European Union Concerted Action involving seven countries and 4537 patients hospitalized for a first-in-a-lifetime stroke, aim to evaluate stroke features, prognosis, and functional outcome in patients with diabetes compared with patients without diabetes [95]. They collected demographic characteristics, traditional risk factors, stroke subtype, clinical manifestation, and outcome. Using logistic regression, the authors examined the statistical impact and the relationship between diabetes and clinical outcome three months after stroke.

**Figure 2:**
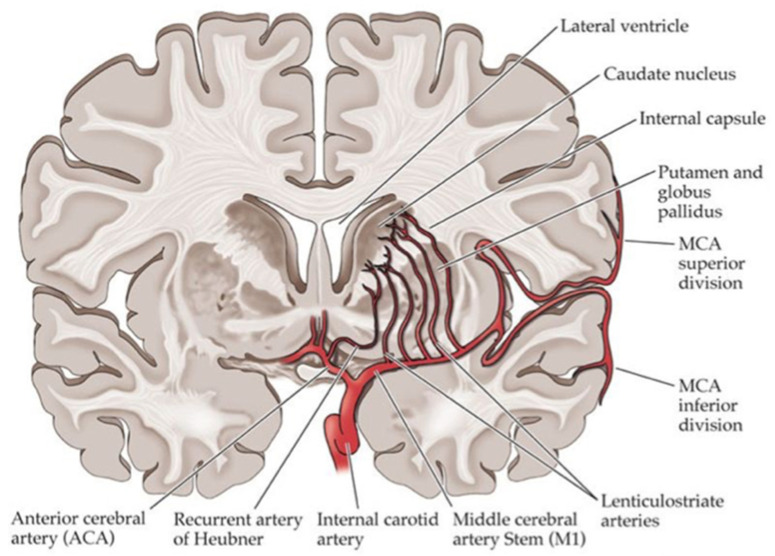
Blood vessels commonly involved in lacunar stroke.

The study results showed that stroke in diabetic patients has a specific clinical pattern and a worse prognosis in terms of disability, defined as Rankin score 2 to 5, and especially in motor function, but no differences were observed in terms of fatality. Diabetic patients had a higher prevalence of ischemic stroke (IS) and a lower prevalence of hemorrhagic stroke (HS). They also described more posterior circulation infarcts (POCI) and lacunar cerebral infarcts (LACI) (Figure 2) than total/partial anterior cerebral infarcts (TACI/PACI). The motor deficit, especially weakness and dysarthria, more common in diabetic patients, may be interpreted as the consequence of small bilateral lesions affecting pyramidal cortico-nuclear tracts through lacunar lesions.

Similarly, Karapanayiotides et al. [90] tried to assess the role of diabetes in stroke patients among 4064 consecutive patients of the Lausanne Stroke Registry. They conducted a multivariate analysis and an adjustment for other risk factors. As a result, they described that diabetes was associated with the lower relative prevalence of intracerebral hemorrhage (ICH), the higher relative prevalence of subcortical infarction (SCI) and higher relative frequency of small-vessel (SVD) and large-artery atherosclerotic stroke (LAAS). One of the possible explanations that the author gave to these results was that the diabetes-induced thickening of the basement membrane and the proliferation of the endothelium leads on the one hand to the small-vessel disease with higher platelet aggregability and coagulability, and on the other hand, would make the cerebral vessels less prone to rupture.

Recently, Larsson et al. [96] conducted a study with the aim to estimate the associations between diabetes and ischemic stroke subtypes among 18,476 ischemic stroke cases of the METASTROKE and the Stroke Genetics Network. The authors showed associations between genetically predicted type 2 diabetes mellitus and large artery stroke and small vessel stroke but not cardioembolic stroke.

Similar findings were documented by Tuttolomondo et al. [77], that diabetes was associated with lacunar ischemic stroke subtype, with a record of hypertension, and with a better Scandinavian Stroke Scale (SSS) score at admission with an association that remained significant even after adjustment for hypertension or large artery atherosclerotic and cardioembolic stroke subtypes.

### Small Vessel Disease in Diabetes Patients

Cerebral small vessel disease (SVD) is a heterogeneous condition including lacunar infarctions, white matter lesions, and cerebral microbleeds that have been shown to be associated with stroke incidence and the development and progression of dementia and diabetic microangiopathy.

The diseases of small cerebral vessels (SVD) have also been recently reclassified into five categories according to the Standards for Reporting Vascular Changes on Neuroimaging (STRIVE) recommendations as follows [101] (Table 2):

- lacunae (formerly defined as silent lacunar infarcts);
- recent small subcortical infarcts (RSSI; formerly categorized as acute lacunar stroke);
- white matter hyperintensity (WMH);
- microbleeds;
- enlarged perivascular space.

The lacunar stroke mechanisms, which cause about 25% of all ischemic strokes, are not yet fully understood.

The hyperglycemic state causes cellular damage by promoting advanced glycation end products, activating protein kinase C, and activating the polyol pathway. Specifically, activating the polyol pathway consumes nicotinamide adenine dinucleotide phosphate, which reduces endothelial nitric oxide synthase activity and decreases nitric oxide production, leading to endothelial dysfunction. Furthermore, by increasing the expression of adhesion molecules in the endothelium and decreasing the anti-inflammatory and vasodilatory effects, this is thought to promote atherosclerosis, leading to the formation of thrombi and consequent occurrence and progression of cerebral infarcts [102,103,104]. The hypothesis of endothelial dysfunction is corroborated by a recent study conducted with the aim to determine if there is evidence of endothelial dysfunction in patients with SVD determining serum levels of cytokines related to endothelial damage [105]. Authors have found increased endothelial soluble markers such as plasma intercellular adhesion molecule 1, thrombomodulin, and tissue factor pathway inhibitor in patients with isolated lacunar stroke and those with additional leukoaraiosis compared with age-matched normal controls. However, it is unclear if these patterns are specific to lacunar stroke because of the absence of non-lacunar controls.

Lacunar infarcts (LIs) are attributed to disease of the penetrating branches of the large cerebral arteries, and the pathological mechanism is thought to mainly involve atherosclerosis secondary to lipohyalinosis, which is caused frequently by hypertension but is also frequently observed in diabetic patients.

Caplan et al. [106] proposed the term ‘branch atheromatous disease’, the cause of which is a microatheroma at the orifice of the penetrating artery. In diabetes, there are relatively many infarcts of this type occurring in the region of the paramedian pontine artery and the region of the lenticulostriate artery branching from the middle cerebral artery [107].

WMLs are typically defined as areas in the cerebral white matter that appear hypodense on computer tomography scans or hyperintense on T2-weighted magnetic resonance imaging (MRI). Depending on the localization within the white matter, these lesions may be classified into two types that are:

- deep WMLs, which are separate from the cerebral ventricle and located in subcortical white matter;
- periventricular WMLs that are connected to the ventricular system.

WMLs are thought to have a vascular origin, they are commonly seen on MR images of neurologically symptomatic elderly people, and due to the increased use of imaging techniques, they are frequently found even in clinically healthy elderly people [108]. In a recent systematic review, Wang et al. [109] observed that most studies described a higher prevalence of WMH among diabetic patients from a macro and microscopic point of view. They also described a more rapid progression of white matter lesions as well as larger lesions.

Mainly periventricular WMLs, rather than subcortical WMLs, are also associated with impairment of cognitive functions, particularly those involving a speed component. Several studies confirmed their association with stroke, of which they may be considered an intermediate surrogate [110]. These lesions increase the rate and risk of stroke in the general population regardless of the other risk factors. Furthermore, this risk increases progressively with the rise of the degree of these lesions, as shown in a study by Kuller et al. in which authors examined the extent of white matter hyperintensity on cranial MRI of 3293 participants from the Cardiovascular Health Study (CHS) and showed that the risk of stroke, in particular lacunar type, is increased significantly as the white matter grade increased [111].

## 6. Effects of Hyperglycemia during Acute Ischemic Stroke

Diabetes mellitus increases the chance of ischemic stroke and is correlated to a worse prognosis in terms of mortality and persistent neurological disability than non-diabetic patients [112,113].

Up to 40% of the subjects hospitalized because of ischemic stroke have high levels of glycemia, typically without a previous diagnosis of diabetes mellitus [114]; furthermore, hyperglycemia rises the mortality due to stroke by 3.3% in non-diabetic patients and is associated with worse neurological disabilities [115].

The processes causing hyperglycemia following acute ischemic stroke are not entirely understood; however, the most accredited hypothesis is the stimulation of the hypothalamohypophyseal-adrenal (HPa) axis by the stress caused by ischemia, resulting in the activation of the sympathetic system and the release of cortisol [116].

Stress hormones promote hyperglycemia, stimulating gluconeogenesis, glycogenolysis, and lipolysis [117,118].

Consequently, hyperglycemia during ischemic stroke could reflect an increased release of catecholamines and cortisol; therefore, according to this hypothesis, the higher the glycemia is, the higher the stroke’s severity [119,120].

In addition, stroke is characterized by the development of inflammation [121] and some proinflammatory cytokines, such as tumor necrosis factor (TNF), seem able to activate the HPa axis [122] and induce insulin resistance [123,124].

During acute ischemic stroke, hyperglycemia seems capable of promoting inflammation and oxidative stress [125], reducing the blood flow to the ischemic penumbra [126], encouraging the entrance of calcium into cerebral cells via *N*-methyl-D-aspartate (NMDA) receptor [127], and altering the metabolic processes in the cerebral parenchyma [128]; all these mechanisms worsen the prognosis.

Hyperglycemia has been associated with overexpression of nuclear factor κB (NF-κB) and reduction of its inhibitor κB (IκB) [125]. NF-κB is a cytoplasmatic transcription factor that is usually bound to IκB; during inflammation, IκB is degraded, making NF-κB migrate to the nucleus [129]. This process promotes the expression of proinflammatory cytokines, such as monocyte chemoattractant protein (MCP-1) and tumor necrosis factor-α (TNF-α), encouraging leucocytes to reach the cerebral parenchyma [130,131]. Furthermore, the presence of glucose is correlated to the overexpression of metalloproteinases (MMP), especially MMP-9 [132], able to damage the blood–brain barrier (BBB), causing further inflammation and oedema [133]. In addition, MMP-9 takes part in central spreading depression organised depolarization of glial cells and neurons that can worsen cerebral edema following ischemic stroke [134].

As reported by Mohanty et al., hyperglycemia causes the activation of p47phox, which is part of NADPH oxidase, resulting in increased levels of superoxide radicals. This process is harmful to the cerebral cells; in fact, it causes carbonylation of the proteins, lipid peroxidation and direct injury of the DNA [135]. In addition, these radicals can also inhibit the release of nitric oxide, resulting in a further decrease of the blood flow to the ischemic area and its penumbra.

Since glucose can promote the expression of the tissue factor (TF) [132] and the plasminogen activator inhibitor-1 (PAI-1), which can encourage thrombosis, hyperglycemia could be involved in the dysfunction of the cerebral microcirculation and could alter the balance between fibrinolysis and thrombosis.

The cerebral vasodilatation stimulated by CO2 is defective in diabetic subjects [136]. This mechanism is mediated mainly by the endothelial release of NO, whose levels are reduced in diabetes. In addition, hyperglycemia promotes the release of reactive species of oxygen (ROS), which can inhibit NO production, leading to further vasoconstriction. Duckrow et al. reported a 24% drop in cerebral blood flow during induced stroke in hyperglycemic mice [137]. Furthermore, Kawai et al. observed a decrease in blood flow in the ischemic penumbra in hyperglycemic rats following obstruction of the middle cerebral artery [126].

Ischemic stroke is characterized per se by increased levels of glutamate, but in animal models, hyperglycemia during cerebral ischemia was correlated to higher extracellular glutamate levels than controls [127]; the excessive presence of this amino acid can unnecessarily activate some receptors, such as the NMDA ones. This process causes an undue flow of calcium into cells provoking mitochondrial damage and the consequent apoptosis. Furthermore, as reported by Araki et al., hyperglycemic cats showed increased intracellular calcium for a more extended period than normoglycemic controls after temporary occlusion of the middle cerebral artery, leading to a worse prognosis [138].

Glycemia seems to influence cellular metabolism: in fact, Chew et al. observed increased cerebral lactic acid and decreased phosphates during stroke in hyperglycemic cats than controls [139]. The metabolic difference between normoglycemic and hyperglycemic subjects is not significant in the ischemic area but is relevant in the penumbra [128]. Excessive lactic acid levels harm the mitochondrial homeostasis, leading to acidosis and the consequent cellular dysfunction and further oedema, compromising neurological recovery [140].

Multiple research demonstrated the protective role of insulin during acute ischemic stroke [141,142]; in fact, it has an anti-inflammatory and anti-thrombotic function and contributes to maintaining the blood flow to the ischemic areas. Insulin can inhibit the multiple pro-inflammatory molecules, such as NF-κB and activator protein 1 (AP-1) [143] and decrease the production of ROS; in addition, insulin impedes the migration of immune cytotypes to the cerebral parenchyma, reducing the plasma levels of intercellular adhesion molecule-1 (ICAM-1) and monocyte chemoattractant protein-1 (MCP-1) [144].

As reported by Aljada et al., insulin plays an anti-thrombotic role by decreasing the expression of TF and PAI-1 [145]; furthermore, it shields the endothelial integrity reducing the release of vascular endothelial growth factor (VEGF) and MMP-9 levels, which injure the blood–brain barrier [146].

Insulin also has an anticoagulant and antiplatelet function: in fact, it reduces the quantity of free fatty acids, which interfere with prostacyclin functioning [147] and promotes the endothelial production of NO, which is involved in vasodilatation and obstacles the platelet aggregation [148].

## 7. Diabetic Foot Syndrome: A Burden of Cardiovascular and Cerebrovascular Risk in Diabetic Patients

Foot ulcerations represent the vascular complications of DM and are associated with a high risk of mortality, morbidity, and disability. They are the leading cause of hospitalization and amputation in diabetic patients, and their management involves spending about 20–40% of healthcare resources [149].

According to World Health Organization, it is possible to encompass all foot complications in the term diabetic foot syndrome (DFS), which is defined as “ulceration of the foot (distally from the ankle and including the ankle) associated with neuropathy and different grades of ischemia and infection” [150]. It, therefore, constitutes a long-term complication of DM that worsens the prognosis and leads to a reduction in the quality of life.

The lifetime risk of a foot ulcer for type 1 or 2 diabetes patients may be as high as 34% [151,152]. Diabetic foot ulcers are a significant cause of morbidity, accounting for at least two-thirds of all nontraumatic amputations performed in the United States [153].

Several population-based studies suggest that the annual cumulative incidence of diabetic foot ulcers varies from 3% to 5% [154]. The prevalence of foot ulcers reported for various populations ranges from 2% to 10% [155]. In a retrospective US cohort study of 8.905 patients with type 1 and type 2 diabetes, the incidence of DFS was 5.8% over an observational period of 3 years [156]. In 15.6% of patients with DFS, lower-limb amputations were necessary, survival was significantly shortened, and the cost for 40–65-year-old males with new foot ulcers was $27,987 for the two years after diagnosis.

Diabetic foot ulcers have multifactorial pathogenesis. In the diabetic patient, various elements—including peripheral neuropathy, foot deformity, abnormal foot pressures, limited joint mobility, external trauma, peripheral artery disease (PAD), and peripheral oedema—act in a common pathway that leads to foot complications. Among these, the leading cause of diabetic ulcers is represented by diabetic peripheral neuropathy (DPN). DPN significantly damage nerve activity throughout the body, impairing motor and sensory function [157]. In sensory neuropathy, the lack of protective sensation makes the foot vulnerable to unattended minor injuries caused by an excess of pressure and mechanical or thermal injury. A critical prospective multicenter study reported that the most frequent component in the pathogenetic axis leading to ulceration in diabetic patients is sensory neuropathy [158].

Other forms of neuropathy can also exert a role in foot ulceration. For example, motor neuropathy injuries the balance of biomechanical forces and the foot anatomy causing foot deformities, limited joint mobility, and compromised loading of the extremities. These pathogenetic disarrangements can also impair the distribution of mechanical forces during walking leading to callus formation, a reactive thickening of the skin, at sites of abnormal load. Tissues close to the callus are more susceptible to ischemic necrosis resulting in loss of skin and subcutaneous tissue and neuropathic ulcer formation representing the final step of this pathogenetic way.

Last but not least, autonomic neuropathy may alter the skin integrity predisposing to dryness and fissuring, creating a potential portal of entry for bacteria. Furthermore, autosympathectomy with consequent sympathetic failure and microvascular thermoregulatory dysfunction damages normal tissue perfusion and microvascular responses to injury.

Another condition that plays a crucial role in the pathogenesis of foot ulcers is PAD, which affects small and large blood vessels. Both macro and microvascular injuries underlying peripheral vascular disease may contribute to the ischemic limb’s inability to heal properly. PAD has an increased incidence and prevalence in subjects with diabetes concerning age and disease duration. Other risk factors generally present in the diabetic population and with a demonstrated role in the pathogenesis of PAD are dyslipidemia, hypertension, and tobacco smoking.

Studies have shown that peripheral vascular disease develops at a younger age among patients with diabetes than the general population [159].

Calculation of the ankle-brachial index (ABI) is a relatively inexpensive and straightforward method to confirm the clinical suspicion and evaluate the severity of PAD [160]. The higher resting systolic blood pressure at the ankle is compared with the higher systolic brachial pressure, and the ratio of the two pressures defines the ABI.

For patients with PAD, the ABI measures the severity of the disease and is predictive of coronary artery disease (CAD) and cerebrovascular disease [161]. In patients with an ABI < 0.90, the relative risk has been reported to be 1.25 (95% CI 1.05, 1.47) for developing an ulcer versus diabetic patients with a normal ABI [159]. In more than 35% of cases, peripheral ischemia due to proximal arterial disease is a significant cause that predisposes to ulceration [162]. Moreover, a comparative study of PAD [159] reported that diabetic patients with PAD are more likely than nondiabetic subjects to have a distal occlusive arterial disease and a higher incidence of amputations and death related to cardiovascular causes. The ischemic foot is characterized by the red color and dryness of the skin and presents clinical findings suggestive of peripheral neuropathy. It is also susceptible to pressure damage from, for example, footwear. Thus, the diabetic foot with ulcerations represents the result of the complex interaction between different pathogenetic factors. Among these, an initial role is played by neuropathy and ischemia, most often together as “neuro-ischemia’, that is, peripheral neuropathy and vascular disease in overlapping, whereas infection is often a consequence. The classic pathophysiological classification of the diabetic foot includes ischemic diabetic foot, neuropathic ischemic foot, and infected diabetic foot. Nevertheless, this type of classification in clinical practice may appear too simple since it is possible to distinguish more frequent clinical variants with mixed features called neuro-ischemic diabetic foot. These clinical variants of DFS have typical morphologic and clinical findings.

The impact of the diabetic foot on public health is significant. Understanding this aspect has led to the development of prevention programmes to raise awareness of the problem in diabetic patients. The purpose is to get them to make lifestyle changes to reduce the likelihood of developing the condition, which unfortunately has no cure. Self-care and health education programs have indeed proved to be effective: several authors have shown that making patients aware of the possibility of developing this complication of T2D and following them over time through the follow-up reduces the possibility of developing chronic ulcers and their progression; conversely, patients from low socio-economic status, with poor income, worse eating habits and worse glycemic control have a very high probability of developing diabetic foot. Prevention measures must not be limited only to modifying dietary habits and lifestyle, but rather to providing practical prevention suggestions relating, for example, to the type of footwear to be used, wound dressing, foot cleansing, and infection management. In addition to this, it is crucial for the doctors to carefully examine the patient at risk, carry out instrumental investigations and direct the patient with lesions at an early stage to an appropriate course of treatment. Ultimately, the management of the diabetic foot is multidisciplinary team management, in which each figure carries out his or her task specifically but in harmony with the other professionals [163].

The chronic management of ulcers in diabetic patients is complex; although science is researching new therapeutic strategies, there are still no drugs that can resolve this condition, resulting from vascular, neurological, and metabolic alterations. In order to have models on which to study this phenomenon, several researchers have used small animals (rabbits, rodents) and larger animals (pigs) with diabetes; progress has undoubtedly been made, but there is still a long way to go, and the most promising path seems to be that of prevention [164].

### 7.1. Cardiovascular Morbidity and DFS

Diabetes is the primary cause of end-stage renal disease, non-traumatic limb amputations, blindness and cardiovascular morbidity and mortality. In addition, mortality in diabetic patients is higher than in nondiabetic subjects.

It is well documented that cardiovascular mortality and morbidity rates are 2–4 times higher among diabetic patients than non-diabetic ones. Roper et al. conducted a study [97] to investigate the age and sex-specific mortality of people with diabetes compared to those without diabetes. Authors reported that diabetes is associated with excess mortality, and the leading causes of death in these patients were ischemic heart disease, cerebrovascular disease, and renal disease. Furthermore, several studies suggest that mortality is higher in the presence of foot ulcers [159,165]. On this basis, Pinto et al. [98] evaluate the possible role of the diabetic foot as a cardiovascular risk marker in type 2 diabetic patients. They enrolled 102 consecutive subjects with type 2 diabetes mellitus with diabetic foot and 123 patients with type 2 diabetes mellitus without foot complications to compare the prevalence of main cardiovascular risk factors, subclinical cardiovascular disease, previous cardiovascular morbidity, and incidence of new vascular events on a 5-year follow-up. The authors reported a higher prevalence of major cardiovascular risk factors (such as dyslipidemia), of asymptomatic markers of cardiovascular disease (CVD), and a higher prevalence and incidence of previous and new-onset vascular events (coronary artery disease, transient ischemic attack/ischemic stroke, diabetic retinopathy) in diabetic patients with foot complications.

At multivariate analysis, duration of diabetes, age, hemoglobin A1c, and DFS maintained a significant association with cardiovascular morbidity; but DFS presence showed the highest hazard ratio.

Furthermore, these authors reported that diabetic patients with lower limb complications showed a higher prevalence of previous cerebrovascular events (transient ischemic attack, ischemic stroke) and incidence of new-onset cerebrovascular events at a 5-year follow-up [166]. Lacunar and large artery atherosclerosis were the most common subtypes underlying the possible pathogenetic importance of the cerebrovascular disease, either atherosclerotic and microvessel disease in patients with diabetic foot.

Based on the results of these studies, DFS could be considered an independent risk factor of morbidity in diabetic subjects.

Considering the reported link between DFS and cardiovascular risk and consistent with the role of putative cardiovascular surrogate markers of arterial stiffness and endothelial function indexes, Tuttolomondo et al. [99] aimed to assess whether DFS is associated with arterial stiffness and endothelial function index impairment. Therefore, they studied 50 subjects with type 2 diabetes mellitus and DFS compared to 50 diabetic subjects without diabetic foot and 53 patients without diabetes mellitus utilizing the mini-mental state examination (MMSE) to evaluate cognitive performance. In addition, carotid-femoral pulse wave velocity (PWV) and augmentation index (Aix) were also evaluated by applanation tonometry (SphygmoCor version 7.1), and the RH-PAT data were digitally analyzed online by Endo-PAT2000 using reactive hyperemia index (RHI) values.

Patients with DFS showed higher mean values of PWV, lower mean values of RHI, and lower mean MMSE. These findings suggest that the presence of DFS is associated with a significant degree of vascular impairment.

The studies mentioned above have suggested that patients with DFS have worse cardiovascular and cerebrovascular risk profiles, higher degrees of endothelial dysfunction and arterial stiffness than patients with diabetes without diabetic foot complications. Moreover, it is well documented that diabetic subjects have an alteration in the sympathovagal balance as assessed by heart rate variability (HRV) analysis, which is also related to endothelial dysfunction. To evaluate the degree of alteration of sympathovagal balance in the setting of DFS, Tuttolomondo et al. [100] have performed the HRV analysis in a cohort of patients with DFS and control patients without diabetic foot complications compared with a population of healthy subjects. They reported that DFS patients show a higher degree of activation of the parasympathetic system than diabetic controls and a higher degree of vascular impairment, as indicated by lower RHI values. The authors also documented a negative correlation between RHI and HRV indices. On this basis, they hypothesized that parasympathetic dysfunction might have a possible additive role in the pathogenesis of vascular complications in subjects with DFS.

### 7.2. Immune-Inflammatory Features of DFS

A complex interplay of several inflammatory and metabolic aspects characterizes diabetes with possible deleterious effects on the cardiovascular system. The simplified explanation may be that the inflammatory background related to diabetes enhances insulin resistance, which is strictly linked to obesity, diabetes, hypertension, prothrombotic conditions, and blood lipid disorders. Furthermore, Tuttle et al. [167] reported a higher degree of serum levels of interleukin (IL)-6 and tumor necrosis factor (TNF)-α in diabetic women with and without CVD compared to nondiabetic women, thus suggesting a common inflammatory state in both diabetes and cardiovascular diseases.

It has been well documented that low-grade immune activation may represent a risk factor for type 2 diabetes and its microvascular and macrovascular complications such as CAD and PAD.

Likewise, this immune-inflammatory upregulation could precede the incidence of a DFS because pro- and anti-inflammatory abnormalities could be significant in different phases of wound healing. Thus, it is suggestive to assume that immune-inflammatory damage may injure tissue homeostasis and wound healing, leading to chronic wounds and realizing a complex clinical condition such as DFS.

Weigelt et al. [168] analyzed the immune-inflammatory background in diabetic subjects with and without foot ulcers by evaluating some immune mediators. They documented an increase in circulating levels of inflammatory markers such as C-reactive protein (CRP), IL-6, fibrinogen, macrophage inflammatory protein-1β, and interferon-γ-inducible protein-10 in patients with diabetic foot complications compared to subjects without foot lesions.

Jeffcoate et al. [150] reported that diabetic foot pathogenesis is characterized by an inflammatory cascade that includes high serum levels of inflammatory cytokines such as TNF-α and IL-1β.

Thus, it is very suggestive that a pronounced inflammatory milieu characterizes DFS.

On this basis, assuming the existence of an interaction between hormones, cytokines, and resistin, Tuttolomondo et al. [169] have evaluated plasma levels of adiponectin, resistin and IL-6 in a cohort of subjects with diabetic foot complications in comparison with patients without foot ulcers.

The study results showed higher IL-6 and resistin plasma levels and lowered adiponectin plasma levels in the cohort of DFS subjects. Although postulated to contribute to insulin resistance, Resistin may contribute to inflammatory responses [170,171]. In contrast with resistin, adiponectin has a documented protective role in cardiovascular disease [172,173,174,175,176,177,178,179,180,181,182,183]. Thus, these findings suggest the importance of inflammatory and metabolic milieu, such as cytokines and adipose hormones in subjects with diabetic foot complications and furtherly underline the burden of risk related to DFS.

## 8. Conclusions

Vascular diseases, particularly atherosclerosis, are undoubtedly the leading causes of disability and death in patients with diabetes mellitus. Diabetes mellitus significantly increases the risk of developing coronary, cerebrovascular, and peripheral arterial disease. The pathophysiology of vascular disease in diabetes involves abnormalities in the endothelial, vascular smooth muscle cell, and platelet function. The metabolic abnormalities characterizing diabetes—such as hyperglycemia, increased free fatty acids, and insulin resistance—provoke molecular mechanisms that contribute to vascular dysfunction. These mechanisms include decreased NO bioavailability, increased oxidative stress, disturbances of intracellular signal transduction, and activation of receptors for AGEs. In addition, platelet function is abnormal, and there is increased production of several prothrombotic factors. These abnormalities contribute to the cellular events that cause atherosclerosis and subsequently increase the risk of adverse cardiovascular events in patients with diabetes, such as ischemic stroke.

The strict association between cerebrovascular diseases and ischemic stroke has been widely demonstrated, and the burden of stroke among diabetic patients is considerable in terms of both mortality and morbidity. Therefore, despite the remarkable pharmacological and technical progress, especially in the acute phase of ischemic stroke, the demand for treatments that can reduce the risk of stroke occurrence and have a neuroprotective effect has dramatically increased.

Diabetes determines the onset of stroke and influences its prognosis by several mechanisms. The effect of this disease on atherosclerosis and the formation of plaques is well documented. However, numerous studies highlight the effect of this disease on the regulation of the microcirculation by altering the production of mediators of cerebral vasomotion and modulating the neuroinflammation. A better understanding of the mechanisms by which hyperglycemia and diabetes exert their harmful effect on the onset and prognosis of stroke may unmask new strategies to reduce cerebrovascular morbidity and mortality in patients with diabetes.

However, in the last few years, a growing body of evidence shows that new antidiabetic drugs are safe and effective in controlling blood glucose and exert several effects that interfere with the molecular mechanisms that underlie vascular damage in diabetes. Like a ‘butterfly effect’, these effects could limit the consequences on the cerebral circulation and the neurological alterations that result from ischemic stroke.

Therefore, future studies are needed to precisely define the extent of the cerebrovascular risk reduction induced by the new antidiabetic drugs, thus opening new scenarios on the management of diabetic patients with stroke.

## Figures and Tables

**Table 1:** Main studies that have evaluated the association between diabetes (or diabetic foot syndrome) and stroke (or preclinical conditions of increased stroke risk).

Main Author	Study Design	Results	Ref.
Framingham study	Cohort study. 20 years of cohort surveillance	2.5-fold incidence of ischemic stroke in diabetic men and a 3.6-fold one in diabetic women.	[74]
Tuttolomondo	Case-control prospective study	Diabetes was associated with lacunar ischemic stroke subtype, with a record of hypertension, and a better Scandinavian Stroke Scale score at admission. The association of diabetes with lacunar stroke remained significant even after adjustment for hypertension or large artery atherosclerotic and cardioembolic stroke subtypes.	[77]
Karapanayiotides	Case-control study	Diabetes was associated with a higher relative frequency of small-vessel and large-artery disease, a lower relative prevalence of intracerebral hemorrhage, a higher relative prevalence of subcortical infarction.	[90]
Manolio	Prospective study	Odds ratio (OR) of 2.12 in those who have diabetes after an adjustment for other risk factors.	[93]
Giles	Prospective study	Odds ratio (OR) of 2.47 in those who have diabetes after an adjustment for other risk factors.	[94]
Megherbi	Prospective study	Diabetic patients, compared with those without diabetes, were more likely to have limb weakness, dysarthria, ischemic stroke, and lacunar cerebral infarction.	[95]
Larsson	Mendelian randomization (MR) analysis	MR analysis showed associations between genetically predicted T2D (type 2 mellitus diabetes) and large artery stroke (OR 1.28) and small vessel stroke (OR 1.21) but not cardioembolic stroke.	[96]
Roper	Longitudinal, population-based study	All-cause standardized mortality ratios for type 2 diabetes were 160 (147 to 174) in women and 141 (130 to 152) in men. Cause-specific standardized mortality ratios were increased for ischemic heart disease, cerebrovascular disease, and renal disease.	[97]
Pinto	Case-control prospective study	Higher prevalence of major cardiovascular risk factors (such as dyslipidemia), of asymptomatic markers of cardiovascular disease (CVD), and a higher prevalence and incidence of previous and new-onset vascular events (coronary artery disease, transient ischemic attack/ischemic stroke, diabetic retinopathy) in diabetic patients with foot complications.	[98]
Tuttolomondo	Case-control study	Patients with DFS (diabetic foot syndrome) showed higher mean values of PWV (pulse wave velocity), lower mean values of RHI (reactive hyperemia index), and lower mean MMSE (mini-mental state examination).	[99]
Tuttolomondo	Case-control study	DFS patients show a higher degree of activation of the parasympathetic system than diabetic controls and a higher degree of vascular impairment, as indicated by lower RHI values.	[100]

**Table 2:** SVD radiological features.

Subtypes of SVD Neuroradiological Aspect	
Recent small subcortical infarct	• Recent infarction in one perforating arteriole and its territory • Increased DWI, FLAIR, T2-weighted signal • Decreased T1-weighted signal • Iso-intense T2-weighted GRE signal
WMHs	• Increase intensity or hyperintensity on T2-weighted, T2 weighted GRE and FLAIR signal • Iso-intense on DWI and T1-weighted signal • Decrease intensity or hypointense on T1-weighted signal
Lacune	• Round or ovoid fluid filed cavity mostly in subcortical region • Hyperintensity on T2-weighted signal • Decreased signal in FLAIR and T1-weighted images • Signal similar to CSF • Decreased or iso-intense signal on DWI
Perivascular space	• Fluid-filled spaces that follow the typical course of a vessel as it goes through gray or white matter similar signal intensity with CSF • Decrease FLAIR and T1-weighted signal • IncreasedT2-weighted-signal • DW1 and T2- weighted GRE signal seems iso-intense
Cerebral microbleed	• Small, rounded areas of signal void • Iso-intense DWI, FLAIR, 12- and-weighted signal • Best seen in T21 weighted GRE with decreased signal

SVD: small vessel disease; WMHs: white matter hyperintensities; DWI: diffusion weighted imaging; FLAIR: fluid attenuated inversion recovery; GRE: gradient echo; CSF: cerebrospinal fluid.
